# On (Non-)Monotonicity and Phase Diagram of Finitary Random Interlacement

**DOI:** 10.3390/e23010069

**Published:** 2021-01-04

**Authors:** Zhenhao Cai, Yunfeng Xiong, Yuan Zhang

**Affiliations:** School of Mathematical Sciences, Peking University, Beijing 100871, China; caizhenhao@pku.edu.cn (Z.C.); xiongyf@math.pku.edu.cn (Y.X.)

**Keywords:** finitary random interlacement, percolation phase transition, critical value

## Abstract

In this paper, we study the evolution of a Finitary Random Interlacement (FRI) with respect to the expected length of each fiber. In contrast to the previously proved phase transition between sufficiently large and small fiber length, for all d≥3, FRI is NOT stochastically monotone as fiber length increases. At the same time, numerical evidence still strongly supports the existence and uniqueness of a critical fiber length, which is estimated theoretically and numerically to be an inversely proportional function with respect to system intensity.

## 1. Introduction

Phase transition, which qualitatively characterizes the change in the state of a system under a continuous change in an external parameter, is ubiquitously found in probabilistic models and statistical mechanics. In this paper, we investigate the phase transitions in the Finitary Random Interlacement (FRI) introduced by Bowen in his study on Gaboriau–Lyons problem [1]. In contrast to its profound connection with the von Neumann–Day problem, a relatively simple description of FRI is given by Bowen in [1] as follows: Consider a random network (G,V) in $Z^{d},\;d\geq 3$. For each vertex x∈V, there lives $N_{x}$ frogs, where $N_{x}$ is a Poisson random variable with mean $u\deg_{x}/\left(T+1\right)$, $\deg_{x}$ is the degree of *x* and u,T are two positive parameters. Each frog has a coin that lands on head with probability T/(T+1). At time t=0, the frog flips the coin. If it lands on heads, the frog moves to a random neighboring vertex with equal probability. It repeats this operation until the coin lands on tails, at which point the frog stops forever. The FRI is the random multiset of random walk paths of all frogs. It is worth noting that *u* gives a natural parameterization of the ”vertex intensity“ of the FRI, as it is proportional to the expected number of vertices visited by all frogs starting from each given point.

Since each path consists of a simple random walk for *t* steps and a geometric random variable with mean T+1 at t+1 steps, a FRI can be roughly treated as a random network (G,V) in $Z^{d}$ “interlaced” by fibers made of geometrically truncated simple random walk (SRW) trajectories, with a multiplicative parameter *u* controlling its Poisson intensity and truncation parameter *T* that determines the expected length of each fiber. As pointed out by an anonymous referee (of a previous paper), an FRI can also be described as a variant of the Random Interlacement (RI) [2] in $Z^{d}$ with weight [3], determined by capacity with a discrete killing measure [4]. See Section 2 for more precise definitions and constructions for FRI.

In the following, we denote by ${\mathrm{FI}}_{d}^{u,T}$ the FRI in $Z^{d}$ with multiplicative parameter *u* and truncation parameter *T*, which is the collection of edges traversed by ”fibers“ in a Poisson point process. See Section 2.1 for details. A key character of the FRI is percolation property, i.e., the existence and uniqueness of an infinite cluster within ${\mathrm{FI}}_{d}^{u,T}$. In contrast to RI, where $I_{d}^{u}$ almost surely percolates for all *d* and u>0, FRI has been proved in [5] to have the following phase transition as an edge percolation model:

- Supercritical phase (Theorem 1, [5]): for all d≥3 and u>0, there is a $0<T_{1}\left(u,d\right)<\infty$ such that for all $T>T_{1}$, ${\mathrm{FI}}_{d}^{u,T}$ almost surely percolates.
- Subcritical phase (Theorem 2, [5]): for all d≥3 and u>0, there is a $0<T_{0}\left(u,d\right)<\infty$ such that for all $0<T<T_{0}$, ${\mathrm{FI}}_{d}^{u,T}$ has no infinite cluster almost surely.

**Figure 1:**
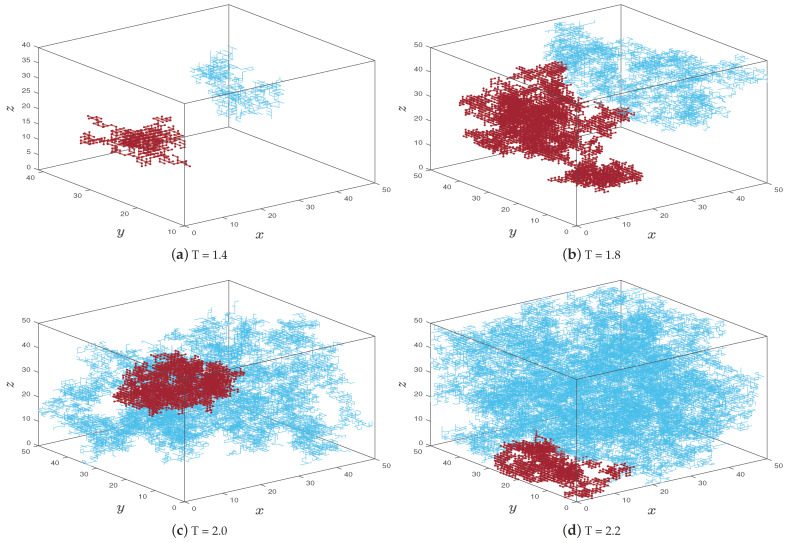
Illustrations of Finitary Random Interlacement (FRI) truncated in a box ${\left[0,50\right]}^{3}$: The first and second largest clusters under u=1/6 and different parameter *T*’s. The sub- and supercritical phases are demonstrated under T=1.4 and T=2.2, respectively. The simulations under T=1.8 and T=2.0 provide some evidence on the percolation phase transition. (**a**) T = 1.4; (**b**) T = 1.8; (**c**) T = 2.0; (**d**) T = 2.2.

Intuitively, the percolation can be visualized by running one realization under different parameters u,T and plotting the first and second largest clusters restricted in a finite box ${\left[0,50\right]}^{3}$. Two small clusters in Figure 1a, that corresponds to u=1/6,T=1.4, provide some evidence that no infinite cluster exists, while a huge cluster along with a smaller cluster in Figure 1d that corresponds to u=1/6,T=2.2 indicates that there may exist only one infinite cluster. One can see that the phase transition may occur near T=1.8, in which the first and second largest clusters are almost of the same size. The dominance of the first largest cluster can be apparently observed as *T* becomes larger, e.g., T=2.0.

Moreover, a follow-up work [6] proved recently that for all d≥3 and u>0, there is a $T_{2}\left(u,d\right)\in \left[T_{1}\left(u,d\right),\infty \right)$ such that for all $T>T_{2}$, the chemical distance on ${\mathrm{FI}}_{d}^{u,T}$ is asymptotically of the same order as the Euclidean distance. Reference [6] further proves that FRI has local uniqueness property for all sufficiently large *T*. See [7] for precise definitions for chemical distance and local uniqueness. However, since ${\mathrm{FI}}_{d}^{u,T}$ may be nonmonotonic with respect to *T*, the existence of a subcritical and a supercritical phase is insufficient to guarantee a critical value in between. It is conjectured in [5] that there is a unique critical value $T_{c}\left(u,d\right)$ such that ${\mathrm{FI}}_{d}^{u,T}$ percolates when $T>T_{c}$ and has no infinite cluster almost surely when $T<T_{c}$.

The percolation phase transition is closely related to the trade-off mechanism with respect to the parameter *T*: As *T* increases, there will be on average fewer and fewer fibers starting from each vertex. In compensation however, the length of each remaining fiber increases proportionally to *T*, so that we are less likely to see the start or end of any fiber locally. In fact, as T→∞, an FRI increasingly resembles the limiting model (which is the classical RI itself) where all fibers are doubly infinite SRW trajectories. It has been proved in [1] that ${\mathrm{FI}}_{d}^{u,T}\Rightarrow I_{d}^{u}$ under the weak-* topology. With the observation above, it is natural to ask how the FRI, as a random collection of edges, evolves with respect to *T*, or more specifically, whether or not it has stochastic monotonicity [5].

Like random interlacement, FRI is by definition monotonically increasing with respect to its intensity *u*. In contrast, it is shown in the paper that for all u>0, the set of edges covered by an ${\mathrm{FI}}_{d}^{u,T}$ is NOT stochastically monotone in *T* for d≥3. This reveals that the evolution of FRI with respect to *T* might be more nontrivial than previously thought, and makes the characterization of the phase diagram of *T* a more interesting question.

The nonmonotonicity of FRI casts shadows on the conjecture of existence and uniqueness of critical value $T_{c}$. A large-scale parallel computing algorithm is employed to explore the behavior of FRI when the fiber length factor *T* changes in the interval $\left[T_{0},T_{1}\right]$. Our numerical findings strongly suggest that, although no longer monotonic, for any d≥3,u>0, ${\mathrm{FI}}_{d}^{u,T}$ still has a unique critical value $T_{c}$ of percolation. For the shape of the phase diagram, we prove that $u^{-1}$ has to be the correct asymptotic order of $T_{c}$ as u→∞, given that the critical value exists; i.e., we find that there are 0<c<C<∞, such that for all sufficiently large *u*

- ${\mathrm{FI}}_{d}^{u,T}$ has no infinite cluster for all T<c/u;
- ${\mathrm{FI}}_{d}^{u,T}$ has a unique infinite cluster for all T>C/u.

Moreover, for all small *u*, we prove that $T_{c}$ has a polynomial upper bound with respect to *u* such that for all δ>0

- when d=3, $T_{c}\leq O\left(\frac{1}{u^{2+\delta }}\right)$;
- when d≥4, $T_{c}\leq O\left(\frac{1}{u^{1+\delta }}\right)$.

At the same time, our numerical tests also indicate that the phase diagram is inversely proportional to *u*.

The rest of this paper is outlined as follows: in Section 2, we recall the precise definition of FRI, together with some important notations and constructions crucial for our theoretical and numerical discussions; in Section 3 we discuss the nonmonotonicity of the edge set of FRI; in Section 4, we discuss theoretical results about characterization of of phase diagram; our numerical explorations on the phase diagram are presented in Section 5.

## 2. Definitions and Notations

In this section, we recall the precise definition of FRI, together with some important notations and constructions in [1,5,6]. We start with some standard notations for simple random walks. Without causing further confusion, we will use $Z^{d}$ to denote both vertices and (the nearest neighbor) edges in the d—dimensional lattice throughout this paper. Then, for a subgraph $G=\left(V,E\right)\subset Z^{d}$, we call it connected if any $v_{1},v_{2}\in V$ can be connected by a collection of edges in *E*. For 1≤j≤d, let $x_{j}\in R^{d}$ satisfy $x_{j}^{\left(i\right)}={𝟙}_{\left\{i=j\right\}}$, 1≤i≤d. Denote the edges $\left\{0,x_{j}\right\}$ and $\left\{0,-x_{j}\right\}$ by $e_{j}$ and $-e_{j}$. Note that ${\left\{e_{j}\right\}}_{j=1}^{d}$ form the basis of $Z^{d}$. For any subset of vertices $A\subset Z^{d}$, let

$$\begin{array}{cc} \; & \partial^{in}A=\left\{x\in A,\;s.t.\exists y\in A^{c},\;{∥x-y∥}_{1}=1\right\}\\ \; & \partial^{out}A=\left\{x\in A^{c},\;s.t.\exists y\in A,\;{∥x-y∥}_{1}=1\right\} \end{array}$$

be the inner and outer boundary of *A*. We let $B\left(x,n\right),\;x\in Z^{d},\;n\geq 1$ be the $l^{\infty }$ box in $Z^{d}$ centered at *x* of radius *n*, i.e.,

$$B\left(x,n\right)=\{y\in Z^{d},\;∥x-y{∥}_{\infty }\leq n\}.$$

Moreover, we abbreviate B(0,n) by B(n).

In this paper, we denote by ${\left\{X_{n}\right\}}_{n=0}^{\infty }$ a simple random walk (SRW) in $Z^{d}$ starting from $X_{0}$, with its distribution denoted by $P_{X_{0}}\left(\cdot \right)$. Note that for any integer $0\leq n_{0}\leq \infty$, the SRW trajectory ${\left\{X_{n}\right\}}_{n=0}^{n_{0}}$ naturally induces a collection of edges ${\left\{\{X_{i},X_{i+1}\}\right\}}_{i=0}^{n_{0}-1}$. Moreover, for any T>0, let $G_{T}\geq 0$ be a geometric random variable with p=1/(T+1) which is independent to ${\left\{X_{n}\right\}}_{n=0}^{\infty }$. Then we call ${\left\{X_{n}\right\}}_{n=0}^{G_{T}}$ a geometrically killed SRW with parameter *T*, and denote its distribution by $P_{X_{0}}^{\left(T\right)}\left(\cdot \right)$ with the convention $P_{X_{0}}^{\left(\infty \right)}\left(\cdot \right)=P_{X_{0}}\left(\cdot \right)$.

Moreover, we denote by

$$\begin{array}{cc} \; & {\bar{H}}_{d,A}^{\left(T\right)}=\inf\left\{n\geq 0,\;X_{n}^{\left(T\right)}\in A\right\}\\ \; & H_{d,A}^{\left(T\right)}=\inf\left\{n\geq 1,\;X_{n}^{\left(T\right)}\in A\right\} \end{array}$$

the first hitting and first returning times to *A*, with the convention inf∅=∞.

**Remark** **1.** *It is worth noting that $H_{d,A}^{\left(T\right)}\equiv \infty$ when $G_{T}=0$.*

For a finite subset *A* and vertex $x\in Z^{d}$, define the killed escape probability

(1) $$Es_{d,A}^{\left(T\right)}\left(x\right)=P_{x}^{\left(T\right)}\left(H_{d,A}^{\left(T\right)}=\infty \right),$$

together with the killed equilibrium measure

(2) $$e_{d,A}^{\left(T\right)}\left(x\right)=\left(2d\right)\times Es_{d,A}^{\left(T\right)}\left(x\right){𝟙}_{x\in A},$$

and the killed capacity

(3) $${\mathrm{cap}}_{d}^{\left(T\right)}\left(A\right)=\sum_{x\in Z^{d}}e_{d,A}^{\left(T\right)}\left(x\right).$$

We also abbreviate ${\bar{H}}_{d,A}^{\left(T\right)}$, $H_{d,A}^{\left(T\right)}$, $Es_{d,A}^{\left(T\right)}$, $e_{d,A}^{\left(T\right)}$, and ${\mathrm{cap}}_{d}^{\left(T\right)}$ to ${\bar{H}}_{d,A}$, $H_{d,A}$, $Es_{d,A}$, $e_{d,A}$, and ${\mathrm{cap}}_{d}$, respectively, when T=∞.

**Remark** **2.** *Factor 2d in the killed equilibrium measure is added for technical reasons to make FRI converge to RI with the same intensity as T→∞. See [1] for details.*

It is worth noting that for $x\in A\backslash \partial^{in}A$,

(4) $${Es}_{d,A}^{\left(T\right)}\left(x\right)=P\left(G_{T}=0\right)=1/\left(1+T\right).$$

### 2.1. Definition of FRI

According to [5], there are two equivalent definitions of the Poisson point process corresponding to FRI. Denote the set of all finite paths on $Z^{d}$ by $W_{d}^{\left[0,\infty \right)}$. Since $W_{d}^{\left[0,\infty \right)}$ is countable, the measure $v_{d}^{\left(T\right)}=\sum_{x\in Z^{d}}\frac{2d}{T+1}P_{x}^{\left(T\right)}$ is a σ— finite measure on $W_{d}^{\left[0,\infty \right)}$.

**Definition** **1.** *For 0<u,T<∞, the finitary random interlacements ${\mathrm{PFI}}_{d}^{u,T}$ is a Poisson point process on $W_{d}^{\left[0,\infty \right)}$ with intensity measure $uv_{d}^{\left(T\right)}$. The law of ${\mathrm{PFI}}_{d}^{u,T}$ is denoted by $P^{u,T}$.*

**Definition** **2.** *For each site $x\in Z^{d}$, $N_{x}$ is a Poisson random variable with parameter $\frac{2du}{T+1}$. Start $N_{x}$ independent geometrically killed simple random walks starting at x with killing rate $\frac{1}{T+1}$. Then, one may equivalently define ${\mathrm{PFI}}_{d}^{u,T}$ as a point measure on $W_{d}^{\left[0,\infty \right)}$ composed of all the trajectories above from all sites in $Z^{d}$.*

With the Poisson point process ${\mathrm{PFI}}_{d}^{u,T}$ defined as above, we define ${\mathrm{FI}}_{d}^{u,T}$ as the collection of all edges traversed by ${\mathrm{PFI}}_{d}^{u,T}$, which is a random subset of edges in $Z^{d}$.

### 2.2. Configurations within a Finite Set

In fact, given a finite set $K\subset Z^{d}$, the distribution of FRI within *K* can be described precisely. By Lemma 2.2 of [5], if we start $N_{x}∼Pois\left(u*e_{d,K}^{\left(T\right)}\left(x\right)\right)$ independent random walks with distribution $P_{x}^{\left(T\right)}$ for any x∈K (denote all these trajectories by ${\left\{\eta_{i}\right\}}_{i=1}^{N_{K}}$), then ${⋃}_{i=1}^{N_{K}}\eta_{i}\cap K$ has the same distribution as ${\mathrm{FI}}_{d}^{u,T}\cap K$.

### 2.3. Stochastic Dominance and Monotonicity

A sufficient condition for the existence of the critical value $T_{c}$ is the stochastic monotonicity with respect to *T*. More precisely, if for any $T^{\prime }>T$ there is a coupling between ${\mathrm{FI}}_{d}^{u,T^{\prime }}$ and ${\mathrm{FI}}_{d}^{u,T}$ such that ${\mathrm{FI}}_{d}^{u,T}\subset {\mathrm{FI}}_{d}^{u,T^{\prime }}$ almost surely, then $T_{c}$ must exist. Therefore, we need the concept of stochastic dominance to describe the existence of the coupling.

**Definition** **3** (Definition 2.1, Chapter 2 of [8]). *Assume that X is a compact metric space with a given partial order. Say a function f on X is monotone if f(η)≤f(ζ) for any η,ζ∈X, η≤ζ. Then, for two probability measures $\mu_{1},\mu_{2}$ on X, say $\mu_{2}$ stochastically dominates $\mu_{1}$ (written by $\mu_{1}\overset{d}{\leq }\mu_{2}$) if and only if for any monotone function f on X,*

$$\int fd\mu_{1}\leq \int fd\mu_{2}.$$

By Theorem 2.4 in the Chapter 2 of [8], we know that the coupling mentioned above exists if and only if ${\mathrm{FI}}_{d}^{u,T}\overset{d}{\leq }{\mathrm{FI}}_{d}^{u,T^{\prime }}$.

## 3. Nonmonotonicity and Single Edge Density

In this section, we first show that unlike the Random Interlacement, an FRI is not stochastically monotone in *T* for all u>0 and d≥3. The following proof was taught to us by an anonymous referee. Recalling the definition of stochastic monotonicity, to prove/disprove ${\mathrm{FI}}_{d}^{u,T_{1}}\overset{d}{\geq }{\mathrm{FI}}_{d}^{u,T_{2}}$ for all $T_{1}\geq T_{2}$, it is equivalent to verify whether or not for all monotonically increasing functions φ on $Z^{d}$, one always has

$$E\left[\varphi \left({\mathrm{FI}}_{d}^{u,T_{1}}\right)\right]\geq E\left[\varphi \left({\mathrm{FI}}_{d}^{u,T_{2}}\right)\right].$$

In particular, we can first take the test function as the very simple form as follows: for each integer n≥1, define

$$\varphi_{0,n}\left(E\right)={𝟙}_{\exists \vec{e}=\left\{x_{1},x_{2}\right\}\in E,\;s.t.\;\left\{x_{1},x_{2}\right\}\cap B\left(n\right)\neq \emptyset }.$$

i.e., $\varphi_{0,n}\left(E\right)$ stands for the event where at least one edge in *E* traverses B(n).

**Theorem** **1.** *For any d≥3, u>0, FRI does not have stochastic monotonicity with respect to T.*

**Proof.**  Recalling the definition of $\varphi_{0,n}\left(\cdot \right)$, one may define

$$A_{n,d}^{u,T}=\left\{\varphi_{0,n}\left({\mathrm{FI}}_{d}^{u,T}\right)=0\right\}$$

to be the event that the FRI fails to intersect B(n). It now suffices to prove that for any d≥3 and u>0 there exists n(d)≥1 and $1<T_{0}\left(d\right)<\infty$ such that

(5) $$P\left(A_{n,d}^{u,T_{0}}\right)>P\left(A_{n,d}^{u,1}\right).$$

Recalling (3) and the construction in Section 2.3, we have for all T>0,

$$P\left(A_{n,d}^{u,T}\right)=\exp\left(-\frac{T}{T+1}u{\mathrm{cap}}_{d}^{\left(T\right)}\left(B\left(n\right)\right)\right).$$

Thus, we only need to show that

(6) $$2{\mathrm{cap}}_{d}^{\left(T_{0}\right)}\left(B\left(n\right)\right)<{\mathrm{cap}}_{d}^{\left(1\right)}\left(B\left(n\right)\right).$$

   □

The proof of (6) is based on the following well-known estimate on the capacity of a box:

**Lemma** **1** (Proposition 6.5.2 in [9]). *There are constants $c_{1},c_{2}>0$ such that for all R>0,*

$$c_{1}R^{d-2}\leq \mathrm{cap}\left(B(R)\right)\leq c_{2}R^{d-2}.$$

Note that $\lim_{T\to \infty }{\mathrm{cap}}_{d}^{\left(T\right)}\left(B\left(n\right)\right)={\mathrm{cap}}_{d}\left(B\left(n\right)\right)$, then, there is a $T_{0}<\infty$ such that ${\mathrm{cap}}_{d}^{\left(T_{0}\right)}\left(B\left(n\right)\right)\leq 2{\mathrm{cap}}_{d}\left(B\left(n\right)\right)\leq 2c_{2}R^{d-2}$. At the same time, by (4)

$$\begin{array}{cc} {\mathrm{cap}}_{d}^{\left(1\right)}\left(B\left(n\right)\right) & \geq \sum_{x\in B(n-1)}e_{d,B(n)}^{\left(1\right)}\left(x\right)\geq {\left(2n-1\right)}^{d}/2\\ \; & >2c_{2}{\left(2n+1\right)}^{d-2}\geq {\mathrm{cap}}_{d}^{\left(T_{0}\right)}\left(B\left(n\right)\right). \end{array}$$

With Theorem 1, one may also consider the evolution of the FRI density for varying *T*’s. Let $\varphi_{1}\left(E\right)={𝟙}_{e_{1}\in E}$ and thus the ”single edge density“

(7) $$E\left[\varphi_{1}\left({\mathrm{FI}}_{d}^{u,T}\right)\right]=P\left(e_{1}\in {\mathrm{FI}}_{d}^{u,T}\right)\overset{\Delta }{=}p_{d,u}\left(T\right)$$

gives the probability that any single (undirected) edge is traversed by the FRI. By translation invariance, $p_{d,u}\left(T\right)$ is proportional to the expectation of the number of edges traversed by FRI within a given set. The following proposition shows that the density of and FRI is not monotone in *T* for d=3,4, but becomes monotonically increasing for higher dimensions.

**Proposition** **1.** *For any u∈(0,∞), $p_{d,u}\left(T\right)\in C^{1}\left(0,\infty \right)$. Moreover, there are $t_{0}\left(d\right)>0$ and $T_{0}\left(d\right)<\infty$ such that*

- *for any d≥3, $p_{d,u}^{\prime }\left(T\right)>0$ for all $T\in (0,t_{0})$;*
- *for d∈{3,4}, $p_{d,u}^{\prime }\left(T\right)<0$ for all $T\in (T_{0},\infty )$;*
- *there exists $d_{0}=d_{0}\left(u\right)<\infty$ such that $p_{d,u}^{\prime }\left(T\right)>0$ for all T∈(0,∞) and $d\geq d_{0}$.*

**Remark** **3.** *As a direct corollary of Theorem 1 and Proposition 1, one may see that, for sufficiently large d, FRI is not monotone as well with respect to its edge density. We expect this should also hold for all d≥3.*

Before presenting the proof of Proposition 1, we first cite the following useful result directly from Exercise 11.1 [9] on the expected length of excursion given a SRW that returns to where it starts.

**Lemma** **2** (from Exercise 11.1, [9]). *Suppose d≥3 and $Y_{n}$ is a simple random walk in $Z^{d}$ with $Y_{0}=0$ and let $\Gamma =\min\{j>0:\;Y_{j}=0\}$. Then,*

$$P\left(\Gamma =2n\right)≍n^{-d/2},\;n\to \infty .$$

*In particular,*

$$E\left[\Gamma |\Gamma <\infty \right]\left\{\begin{array}{c} =\infty ,\;d\leq 4,\\ <\infty ,\;d\geq 5. \end{array}\right.$$

We also need a ”high dimensional“ version of Lemma 2. The main technicalities involved in the proof are asymptotic estimates for high-dimensional SRW’s, which are not directly related to the main scope of this paper, so we leave it in Appendix A.

**Lemma** **3.** *For d≥5, let*

$$R_{d}=E_{0}\left[H_{d,\{0,x_{1}\}}{𝟙}_{H_{d,\{0,x_{1}\}}<\infty }\right]<\infty .$$

*Then, $\lim_{d\to \infty }R_{d}=0$.*

Now we are able to prove Proposition 1.

**Proof** **of** **Proposition** **1.** We denote the event $\left\{e_{1}\;is\;not\;contained\;by\;the\;trajectory\right\}$ by *F* and then calculate $P^{u,T}\left(e_{1}\notin {\mathrm{FI}}_{d}^{u,T}\right)$. First, we need to calculate $P_{x_{2}}^{\left(T\right)}\left(F\right)$ and $P_{-x_{1}}^{\left(T\right)}\left(F\right)$. Denote that $E_{1}=P_{-x_{1}}^{\left(T\right)}\left(H_{d,\{0,x_{1}\}}^{\left(T\right)}=\infty \right)$ and $E_{2}=P_{x_{2}}^{\left(T\right)}\left(H_{d,\{0,x_{1}\}}^{\left(T\right)}=\infty \right)$. We have

(8) $$\begin{array}{cc} P_{x_{2}}^{\left(T\right)}\left(F\right)= & E_{2}+\sum_{n=1}^{\infty }P_{x_{2}}^{\left(T\right)}\left(H_{d,\{0,x_{1}\}}^{\left(T\right)}=n,F\right)\\ = & E_{2}+\sum_{n=1}^{\infty }P_{x_{2}}^{\left(T\right)}\left(H_{d,\{0,x_{1}\}}^{\left(T\right)}=n\right)\left[\frac{1}{T+1}+\frac{T}{T+1}\left(\frac{2d-2}{2d}P_{x_{2}}^{\left(T\right)}\left(F\right)+\frac{1}{2d}P_{-x_{1}}^{\left(T\right)}\left(F\right)\right)\right]\\ = & E_{2}+\left(1-E_{2}\right)\left[\frac{1}{T+1}+\frac{T}{T+1}\left(\frac{2d-2}{2d}P_{x_{2}}^{\left(T\right)}\left(F\right)+\frac{1}{2d}P_{-x_{1}}^{\left(T\right)}\left(F\right)\right)\right]. \end{array}$$

In the same way, we have

(9) $$P_{-x_{1}}^{\left(T\right)}\left(F\right)=E_{1}+\left(1-E_{1}\right)\left[\frac{1}{T+1}+\frac{T}{T+1}\left(\frac{2d-2}{2d}P_{x_{2}}^{\left(T\right)}\left(F\right)+\frac{1}{2d}P_{-x_{1}}^{\left(T\right)}\left(F\right)\right)\right].$$

Combine (8) and (9),

(10) $$\begin{array}{cc} \; & P_{x_{2}}^{\left(T\right)}\left(F\right)\left[1+\left(E_{2}-E_{1}\right)*\frac{T}{T+1}*\frac{2d-2}{2d}\right]\\ = & P_{-x_{1}}^{\left(T\right)}\left(F\right)\left[1+\left(E_{1}-E_{2}\right)\frac{T}{T+1}*\frac{1}{2d}\right]+\left(E_{2}-E_{1}\right)\frac{T}{T+1}. \end{array}$$

By (8) and (10), we have

(11) $$\begin{array}{cc} \; & P_{-x_{1}}^{\left(T\right)}\left(F\right)*\frac{\left(1-E_{2}\right)*\frac{T}{T+1}*\frac{1}{2d}}{1-\frac{T}{T+1}*\frac{2d-2}{2d}*\left(1-E_{2}\right)}+\frac{E_{2}+\left(1-E_{2}\right)*\frac{1}{T+1}}{1-\frac{T}{T+1}*\frac{2d-2}{2d}*\left(1-E_{2}\right)}\\ = & P_{-x_{1}}^{\left(T\right)}\left(F\right)*\frac{1-\left(E_{2}-E_{1}\right)*\frac{T}{T+1}*\frac{1}{2d}}{1+\left(E_{2}-E_{1}\right)*\frac{T}{T+1}*\frac{2d-2}{2d}}+\frac{\left(E_{2}-E_{1}\right)\frac{T}{T+1}}{1+\left(E_{2}-E_{1}\right)*\frac{T}{T+1}*\frac{2d-2}{2d}}. \end{array}$$

Therefore,

(12) $$\begin{array}{cc} P_{-x_{1}}^{\left(T\right)}\left(F\right)= & \frac{E_{2}+\left(1-E_{2}\right)\frac{1}{T+1}-\left(E_{2}-E_{1}\right)\frac{2}{2d}\frac{T}{T+1}}{1-\left[\frac{2d-1}{2d}\left(1-E_{2}\right)+\frac{1}{2d}\left(E_{2}-E_{1}\right)\right]\frac{T}{T+1}}\\ = & \frac{\left[\frac{2d-2}{2d}E_{2}+\frac{2}{2d}E_{1}\right]T+1}{\left[\frac{2d-2}{2d}E_{2}+\frac{1}{2d}E_{1}+\frac{1}{2d}\right]T+1}. \end{array}$$

In the same way, we have

(13) $$\begin{array}{c} P_{x_{2}}^{\left(T\right)}\left(F\right)=\frac{\left[\frac{2d-1}{2d}E_{2}+\frac{1}{2d}E_{1}\right]T+1}{\left[\frac{2d-2}{2d}E_{2}+\frac{1}{2d}E_{1}+\frac{1}{2d}\right]T+1}. \end{array}$$

Therefore,

(14) $$\begin{array}{cc} P_{0}^{\left(T\right)}\left(F\right)= & \frac{1}{T+1}+\frac{T}{T+1}\left[\frac{2d-2}{2d}P_{x_{2}}^{\left(T\right)}\left(F\right)+\frac{1}{2d}P_{-x_{1}}^{\left(T\right)}\left(F\right)\right]\\ = & \frac{\left[\frac{2d-2}{2d}E_{2}+\frac{1}{2d}E_{1}\right]T+1}{\left[\frac{2d-2}{2d}E_{2}+\frac{1}{2d}E_{1}+\frac{1}{2d}\right]T+1}. \end{array}$$

Restricted on $\left\{0,x_{1}\right\}$, there are $Pois\left(2du*Es_{d,\{0,x_{1}\}}^{\left(T\right)}\left(0\right)\right)$ independent trajectories starting from 0 and $Pois\left(2du*Es_{d,\{0,x_{1}\}}^{\left(T\right)}\left(1\right)\right)$ trajectories starting from 1. Note that $Es_{d,\{0,x_{1}\}}^{\left(T\right)}\left(0\right)=Es_{d,\{0,x_{1}\}}^{\left(T\right)}\left(1\right)$, we have

(15) $$\begin{array}{cc} P^{u,T}\left(e_{1}\notin {\mathrm{FI}}_{d}^{u,T}\right)= & {\left[\sum_{m=0}^{\infty }\exp\left(-2du*Es_{d,\{0,x_{1}\}}^{\left(T\right)}\left(0\right)\right)\frac{{\left(2du*Es_{d,\{0,x_{1}\}}^{\left(T\right)}\left(0\right)\right)}^{m}}{m!}{\left(P_{0}^{\left(T\right)}\left(F\right)\right)}^{m}\right]}^{2}\\ = & \exp(-4du*Es_{d,\{0,x_{1}\}}^{\left(T\right)}\left(0\right)\left(1-P_{0}^{\left(T\right)}\left(F\right)\right)). \end{array}$$

Let $f\left(T\right)=Es_{d,\{0,x_{1}\}}^{\left(T\right)}\left(0\right)=Es_{d,\{0,x_{1}\}}\left(0\right)+\sum_{n=1}^{\infty }P_{0}\left(H_{d,\{0,x_{1}\}}=n\right)\left(1-{\left(1-\frac{1}{T+1}\right)}^{n}\right)$ and $g\left(T\right)=1-P_{0}^{\left(T\right)}\left(F\right)=\frac{\frac{1}{2d}T}{\left[\frac{2d-2}{2d}E_{2}+\frac{1}{2d}E_{1}+\frac{1}{2d}\right]T+1}$. We have

(16) $$\begin{array}{cc} f^{\prime }\left(T\right)= & -\frac{1}{{\left(T+1\right)}^{2}}\sum_{n=1}^{\infty }\left[P_{0}\left(H_{d,\{0,x_{1}\}}=n\right)*n*{\left(1-\frac{1}{T+1}\right)}^{n-1}\right]\\ = & -\frac{1}{T(T+1)}\sum_{n=1}^{\infty }\left[P_{0}\left(H_{d,\{0,x_{1}\}}=n\right)*n*{\left(1-\frac{1}{T+1}\right)}^{n}\right]\\ = & -\frac{1}{T(T+1)}*E_{0}^{\left(T\right)}\left[H_{d,\{0,x_{1}\}}^{\left(T\right)};1\leq H_{d,\{0,x_{1}\}}^{\left(T\right)}<\infty \right]<\infty . \end{array}$$

Meanwhile,

(17) $$\begin{array}{cc} g^{\prime }\left(T\right)= & \frac{\frac{1}{2d}}{{\left[\left(\frac{2d-2}{2d}E_{2}+\frac{1}{2d}E_{1}+\frac{1}{2d}\right)+\frac{1}{T}\right]}^{2}}*\frac{1}{T^{2}}\\ = & \frac{\frac{1}{2d}}{{\left[\left(\frac{2d-2}{2d}E_{2}+\frac{1}{2d}E_{1}+\frac{1}{2d}\right)T+1\right]}^{2}}\overset{\Delta }{=}\frac{\frac{1}{2d}}{{\left(aT+1\right)}^{2}}, \end{array}$$

where $a=\frac{2d-2}{2d}E_{2}+\frac{1}{2d}E_{1}+\frac{1}{2d}$. Combine (16) and (17),

(18) $$\begin{array}{cc} {\left(f\cdot g\right)}^{\prime }\left(T\right)= & Es_{d,\{0,x_{1}\}}^{\left(T\right)}\left(0\right)*\frac{\frac{1}{2d}}{{\left(aT+1\right)}^{2}}-\frac{1}{T(T+1)}\\ & *E_{0}^{\left(T\right)}\left[H_{d,\{0,x_{1}\}}^{\left(T\right)};1\leq H_{d,\{0,x_{1}\}}^{\left(T\right)}<\infty \right]*\frac{\frac{1}{2d}T}{aT+1}\\ = & \frac{\frac{1}{2d}}{{\left(aT+1\right)}^{2}}\left[Es_{d,\{0,x_{1}\}}^{\left(T\right)}\left(0\right)-\frac{aT+1}{T+1}*E_{0}^{\left(T\right)}\left[H_{d,\{0,x_{1}\}}^{\left(T\right)};1\leq H_{d,\{0,x_{1}\}}^{\left(T\right)}<\infty \right]\right]<\infty . \end{array}$$

Therefore, $p_{d,u}\left(T\right)=1-\exp\left(-4du*f\left(T\right)*g\left(T\right)\right)\in C^{1}\left(0,\infty \right)$.

Note that ∀d≥3, $\lim_{T\to 0+}Es_{d,\{0,x_{1}\}}^{\left(T\right)}\left(0\right)=1$, and $\lim_{T\to 0+}E_{0}^{\left(T\right)}\left[H_{d,\{0,x_{1}\}}^{\left(T\right)};1\leq H_{d,\{0,x_{1}\}}^{\left(T\right)}<\infty \right]=0$, we have

(19) $$\lim_{T\to 0+}{\left(f\cdot g\right)}^{\prime }\left(T\right)=\frac{1}{2d}>0.$$

Similar to Lemma 2, for d=3,4, $E_{0}\left[H_{\left\{0,x_{1}\right\}};1\leq H_{\left\{0,x_{1}\right\}}<\infty \right]=\infty$. Then, we have

(20) $$\lim_{T\to \infty }E_{0}^{\left(T\right)}\left[H_{d,\{0,x_{1}\}}^{\left(T\right)};1\leq H_{d,\{0,x_{1}\}}^{\left(T\right)}<\infty \right]=\infty .$$

Note that $\lim_{T\to \infty }Es_{d,\{0,x_{1}\}}^{\left(T\right)}\left(0\right)=Es_{d,\{0,x_{1}\}}\left(0\right)$, we know that $\exists T_{0}>0$ such that $\forall T>T_{0}$,

(21) $$Es_{d,\{0,x_{1}\}}^{\left(T\right)}\left(0\right)-\frac{aT+1}{T+1}*E_{0}^{\left(T\right)}\left[H_{d,\{0,x_{1}\}}^{\left(T\right)};1\leq H_{d,\{0,x_{1}\}}^{\left(T\right)}<\infty \right]<0.$$

For d≥5, it is fundamental to construct a coupling between SRWs on $Z^{d}$ and $Z^{3}$ such that $\left\{H_{d,\{0,x_{1}\}}<\infty \right\}\subset \left\{H_{3,\{0,x_{1}\}}<\infty \right\}$. Thus,

(22) $$Es_{d,\{0,x_{1}\}}^{\left(T\right)}\left(0\right)>Es_{d,\{0,x_{1}\}}\left(0\right)\geq Es_{3,\{0,x_{1}\}}\left(0\right).$$

By Lemma 3, there exists $d_{0}$ such that for any $d>d_{0}$ and T>0,

(23) $$E_{0}^{\left(T\right)}\left[H_{d,\{0,x_{1}\}}{𝟙}_{H_{d,\{0,x_{1}\}}<\infty }\right]<E_{0}\left[H_{d,\{0,x_{1}\}}{𝟙}_{H_{d,\{0,x_{1}\}}<\infty }\right]\leq Es_{3,\{0,x_{1}\}}\left(0\right).$$

By (18), (22), and (23), for any $d>d_{0}$ and 0<T<∞, we have

(24) $${\left[Es_{d,\{0,x_{1}\}}^{\left(T\right)}\left(0\right)\left(1-P_{0}^{\left(T\right)}\left(F\right)\right)\right]}^{\prime }>\frac{\frac{1}{2d}}{{\left(aT+1\right)}^{2}}\left[Es_{3,\{0,x_{1}\}}\left(0\right)-Es_{3,\{0,x_{1}\}}\left(0\right)\right]=0.$$

Recall that $p_{d,u}\left(T\right)=1-\exp\left(-4du*Es_{d,\{0,x_{1}\}}^{\left(T\right)}\left(0\right)\left(1-P_{0}^{\left(T\right)}\left(F\right)\right)\right)$, then the proof is complete.    □

**Remark** **4.** *Though it is true that Lemma 2 as stated is for the expected time of returning to 0 rather than to $\left\{0,x_{1}\right\}$, the result and proof are exactly parallel for returning to any finite subset. So, we decide to cite [9] rather than repeat the proof.*

**Remark** **5.** *Note that Proposition 1 gives an alternative proof for nonmonotonicity when d=3,4.*

**Figure 2:**
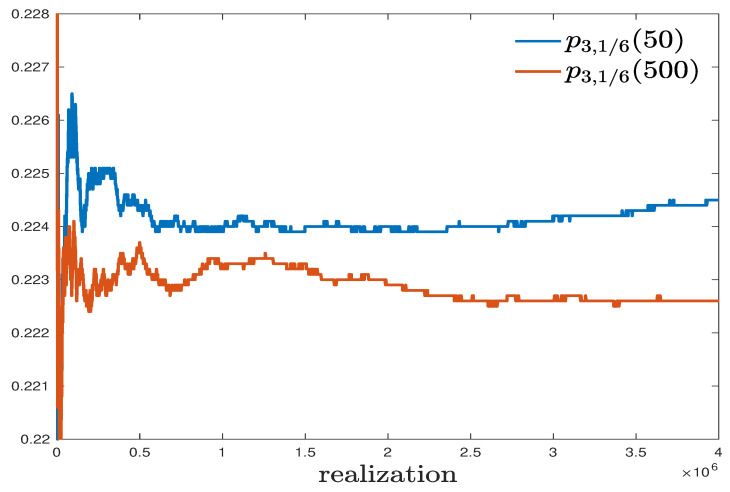
Numerical approximations to $p_{3,1/6}\left(50\right)$ and $p_{3,1/6}\left(500\right)$ by $4\times 10^{6}$ i.i.d. stochastic realizations.

In addition to the aforementioned theoretical proof, the low-dimensional nonmonotonicity can also be verified in numerical simulation. In Figure 2, we present numerical approximations of $p_{3,1/6}\left(50\right)$ and $p_{3,1/6}\left(500\right)$ achieved from $4\times 10^{6}$ i.i.d. stochastic realizations.

In Figure 2, a significant difference between the blue and red curves is observed. With $4\times 10^{6}$ i.i.d. stochastic realizations, we have the frequencies

$$N_{b}\overset{d}{=}B\left(4\times 10^{6},p_{3,1/6}\left(50\right)\right),\;N_{r}\overset{d}{=}B\left(4\times 10^{6},p_{3,1/6}\left(500\right)\right).$$

**Figure 3:**
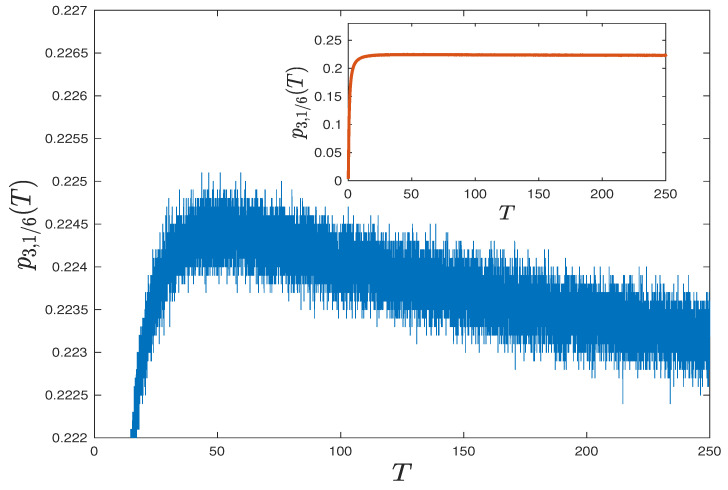
Numerical approximations for $p_{3,1/6}\left(u\right),\;T\in \left[0,250\right]$ with spacing ΔT=0.01. Each point is evaluated by $4\times 10^{6}$ i.i.d. stochastic realizations. The probability seems to be nonmonotonic and reaches its maximum at about $T_{\max}=50$.

So, their standard deviations can be bounded from above by $1/\left(2\times 2\times 10^{3}\right)=2.5\times 10^{-4}$. However, the difference between our approximations is about $1.7\times 10^{-3}$, which is larger than 4 times the upper bound of standard deviation. In Figure 3, we numerically approximate the single edge density $p_{3,1/6}\left(\cdot \right)$ for different *T* with spacing ΔT=0.01, and each point is evaluated by $4\times 10^{6}$ i.i.d. stochastic realizations. In spite of some stochastic fluctuations, the trend of nonmonotonicity is clear and the probability seems to reach maximum at $T_{max}\approx 50$.

## 4. Characterization of Phase Diagram

In this section, we focus on the (potential) phase diagram of edge percolation in FRI. We start with proving the uniqueness of infinite cluster in FRI by the classical “finite energy” argument. We put technical details of the proof in Appendix B.

**Theorem** **2.** *For any d≥3 and u,T>0,*

$$P^{u,T}\left(FRI\;has\;at\;most\;one\;infinite\;cluster\right)=1.$$

**Remark** **6.** *Theorem 2 is similar to, though slightly stronger than, Theorem 4 [5], where the uniqueness was proved for sufficiently large T’s.*

**Theorem** **3.** *For all d≥3 and FRI ${\mathrm{FI}}_{d}^{u,T}$, we have the following:*

1. *If ${\mathrm{FI}}_{d}^{u,T}$ has an infinite cluster almost surely, then so does ${\mathrm{FI}}_{d}^{u^{\prime },T}$ for all $u^{\prime }>u$.*
2. *(Theorem 1, [5]) For all u>0, there is a $0<T_{1}\left(u,d\right)<\infty$ such that for all $T>T_{1}$, ${\mathrm{FI}}_{d}^{u,T}$ almost surely percolates.*
3. *Let $p_{d}^{c}$ be the critical edge density for d—dimensional Bernoulli bond percolation. For any $u>-2\log(1-p_{d}^{c})$, there exist some δ=δ(u,d)>0 such that ${\mathrm{FI}}_{d}^{u,T}$ percolates almost surely for $T\in [{\left(1+\delta \right)}^{-1},1+\delta ]$. Moreover, for any fixed d, δ(u,d)≍u as u→∞.*
4. *For any d≥3, there is $U_{d}<\infty$, such that for all $u\geq U_{d}$, ${\mathrm{FI}}_{d}^{u,T}$ percolates almost surely for all $T\geq {\left(1+\delta \left(u,d\right)\right)}^{-1}$.*
5. *For any 0<δ<1 and $m_{0}>0$, there exists constant $M=M(d,\delta ,m_{0})<\infty$ such that*
  (a) *when d=3, $\forall 0<u\leq m_{0}$, $T>\frac{M}{u^{2+\delta }}$, ${\mathrm{FI}}_{d}^{u,T}$ almost surely percolates;*
  (b) *when d≥4, $\forall 0<u\leq m_{0}$, $T>\frac{M}{u^{1+\delta }}$, ${\mathrm{FI}}_{d}^{u,T}$ almost surely percolates.*

**Remark** **7.** *A statement equivalent to Claim (iii) Theorem 3 has also been proved in 5), Remark V.5.3, [10].*

**Proof.**  Note that by Theorem 2, one may focus only on the existence of infinite cluster.

Claim (i) is an immediate result of the monotonicity of ${\mathrm{FI}}_{d}^{u,T}$ with respect to *u*. For Claim (iii) and (iv), the key idea is to bound ${\mathrm{FI}}_{d}^{u,T}$ from below by a supercritical Bernoulli percolation. Without loss of generality, one may first consider vertex 0, edge $e_{1}$, and the collection of fibers with length ≥1 that start from 0 and traverse $e_{1}$ in their first jump. We denote the number of such paths by $N_{e_{1},+}$. Recalling the definition of ${\mathrm{FI}}_{d}^{u,T}$, there are Pois(2du/(T+1)) fibers starting from 0. While for each of them, the probability it has length at least 1 is T/(T+1), and the probability it takes $e_{1}$ in the first step is ${\left(2d\right)}^{-1}$. Thus, by the thinning property of Poisson distribution, we have

$$N_{e_{1},+}\overset{d}{=}Pois\left(uT/{\left(T+1\right)}^{2}\right).$$

Similarly, one can define $N_{e_{1},-}$ to be the number of fibers that start from $x_{1}$ and traverse $e_{1}$ in their first jump. By independent increment property of PPP, $N_{e_{1},-}$ is independent and identically distributed as $N_{e_{1},+}$. Define the event as

$$\left\{e_{1}\;\mathrm{is}\;\mathrm{good}\right\}\overset{\Delta }{=}\left\{N_{e_{1},+}+N_{e_{1},-}>0\right\}.$$

Moreover, for any edge $e=\left\{x,y\right\}\in Z^{d}$, one can also define $N_{e,+},\;N_{e,-}$ in the exact same way. Thus, $\left\{N_{e,\pm },e\in Z^{d}\right\}$ form a i.i.d. sequence of $\mathrm{Poisson}(uT/{\left(T+1\right)}^{2})$. Once again, define

$$\left\{e\;\mathrm{is}\;\mathrm{good}\right\}\overset{\Delta }{=}\left\{N_{e,+}+N_{e,-}>0\right\}.$$

Thus, the collection of good edges by definition forms a Bernoulli bond percolation with single edge density

(25) $$p=1-P\left(N_{e_{1},+}=0\right)\cdot P\left(N_{e_{1},-}=0\right)=1-\exp\left(-2uT/{\left(T+1\right)}^{2}\right),$$

which percolates when

$$\frac{uT}{{\left(T+1\right)}^{2}}\geq -\log\left(1-p_{d}^{c}\right)/2.$$

Therefore, for any $u>-2\log(1-p_{d}^{c})$, we take $\delta \left(u,d\right)=C\left(u,d\right)-2+\sqrt{C{\left(u,d\right)}^{2}-2C\left(u,d\right)}$, where $C\left(u,d\right)=\frac{u}{-\log(1-p_{d}^{c})}>2$. Note that a good edge is by definition always traversed by the FRI. Claim (iii) is now a direct result of (25), the fact that $T/{\left(T+1\right)}^{2}$ reaches its maximum of 1/4 at T=1, and that $T/{\left(T+1\right)}^{2}≍T^{-1}$ as T→∞.

Now, for (iv), note that for a fixed *u*, say u=1, by Theorem 1 [5], there is a $T_{1}$ such that for all $T>T_{1}$, ${\mathrm{FI}}_{d}^{1,T}$ has an infinite cluster almost surely. With Claim (i), we now know this also holds for all u≥1. In (iii), we showed that δ(u,d)≍u. Thus, there is always a $U_{d}$ such that $\delta \left(u,d\right)\geq T_{1}$ for all $u\geq U_{d}$. Thus, we have an infinite cluster almost surely for all *T* from ${\left(1+\delta \left(u,d\right)\right)}^{-1}$ all the way to infinity.

The proof of (v) is based on some more careful controls of exponents in constructing the infinite cluster in [5]. As it is redundant to repeat the shared part of our construction in full details, we just point out modifications and estimates necessary to our proof here. Note that it is sufficient to prove for 0<δ<0.5.

To be specific, let ϵ be any sufficiently small positive constant and $R=\lfloor T^{0.5+\epsilon }\rfloor$, $r=\lfloor T^{0.5-\epsilon }\rfloor$. By [5], we know that it is sufficient to prove the following three events happen with sufficiently high probability (i.e., larger than $1-p_{0}\left(d\right)$ for some given $p_{0}\left(d\right)$), corresponding to the conditions 1–3 introduced in Definition 3 of [5]:

1. Assume that ${\mathrm{FI}}_{d}^{u,T}$ is the union of two independent FRI copies ${\mathrm{FI}}_{d,1}^{0.5u,T}$ and ${\mathrm{FI}}_{d,2}^{0.5u,T}$ (by the property of Poisson point processes). For any box B(z,r)⊂B(R), there exists a connected cluster $A\subset B(z,r+T^{0.5+0.5\epsilon })$ in ${\mathrm{FI}}_{d,1}^{0.5u,T}$ traversing B(z,r) and $cap\left(A\right)>CT^{\frac{\left(d-2)(1-\epsilon \right)}{2}}$. We denote this event by *E*.
2. For any x,y∈B(R) such that |x−y|≤3r and two connected clusters $C_{x}$ and $C_{y}$ (containing *x* and *y*, respectively) in ${\mathrm{FI}}_{d,1}^{0.5u,T}$, if $cap\left(C_{x}\right)>CT^{\frac{\left(d-2)(1-\epsilon \right)}{2}}$, $cap\left(C_{y}\right)>CT^{\frac{\left(d-2)(1-\epsilon \right)}{2}}$, and $C_{x}\cup C_{y}\subset B\left(2T^{0.5+0.5\epsilon }\right)$, then $C_{x}$ and $C_{y}$ are connected by ${\mathrm{FI}}_{d,2}^{0.5u,T}$ within B(1.4R). We denote this event by *F*.
3. There is no path starting from $Z^{d}\backslash B\left(2R\right)$ and intersecting B(1.5R). In addition, for 1≤j≤d, there is no path starting from $\left\{x\in B\left(2R\right):-2R\leq x^{\left(j\right)}\leq -R\right\}$ and intersecting $\left\{x\in B\left(2R\right):-0.5R\leq x^{\left(j\right)}\leq 1.5R\right\}$, and no path starting from $\left\{x\in B\left(2R\right):R\leq x^{\left(j\right)}\leq 2R\right\}$ and intersecting $\left\{x\in B\left(2R\right):-1.5R\leq x^{\left(j\right)}\leq 0.5R\right\}$. We denote this event by *G*.

 □

First, for event *E*, we need an estimate for the capacity of trajectories of several simple random walks.

**Lemma** **4** (Lemma 5, [11]). *Let ${\left\{X_{i}\right\}}_{i=1}^{N}$ be a sequence of independent simple random walks on $Z^{d}$ and $\Phi \left({\bar{X}}_{N},T\right)=\overset{N}{\underset{i=1}{⋃}}\left\{X_{i}\left(t\right):0\leq t\leq T\right\}$. Then,*

$$P\left[cap\left(\Phi ({\bar{X}}_{N},T)\right)\geq c*\min\left\{N*F\left(d,T\right),T^{\frac{d-2}{2}}\right\}\right]\geq \frac{c}{{\left(\log\left(T\right)\right)}^{2}},$$

*where*

$$F\left(d,T\right):=\left\{\begin{array}{ccc} & T^{0.5},\;\;\;\;\;\; & d=3;\\ & \frac{T}{\log(T)},\;\;\;\;\;\; & d=4;\\ & T,\;\;\;\;\;\; & d\geq 5. \end{array}\right.$$

By Lemma 4 and the same approach mentioned in the proof of Lemma 6 [11], we can get a stronger version of Lemma 6 [11]:

(26) $$P\left(cap\left(\Phi \left({\bar{X}}_{N},T\right)\right)\geq C\min\left\{NF\left(d,T^{1-\epsilon }\right),T^{\frac{\left(d-2)(1-\epsilon \right)}{2}}\right\}\right)\leq 1-\exp\left(-CT^{0.5\epsilon }\right).$$

When d=3, note that the number of paths traversing B(z,r) with length at least *T* is a Poisson random variable with parameter $cu*r^{d-2}=c*ur$. By the large deviation bound for Poisson distribution, the probability of the event that there exists one path traversing B(z,r) with length at least *T* is larger than 1−exp−C∗ur. Take N=1 in (26), then, we have

(27) $$\begin{array}{cc} \; & P\left(\exists a\;path\;\eta \;in\;{\mathrm{FI}}_{d,1}^{0.5u,T}\;traversing\;B\left(z,r\right)\;and\;cap\left(\eta \right)>CT^{\frac{\left(d-2)(1-\epsilon \right)}{2}}\right)\\ \geq & 1-\exp\{-C*ur\}-\exp(-CT^{0.5\epsilon }). \end{array}$$

When d≥4, we do the same construction in Section 4.3 of [5] by using the paths in ${\mathrm{FI}}_{d,1}^{0.5u,T}$ with length at least *T* (note that we will use (26) for $n_{0}+1$ times but not only d−2 times, where $n_{0}=\frac{d}{2(0.5\delta -\epsilon )}$). Similarly, by (26) and large deviation bound for Poisson distribution, we have

(28) $$\begin{array}{cc} & P\left(\exists a\;connected\;cluster\;A\;in\;{\mathrm{FI}}_{d,1}^{0.5u,T}\;traversing\;B\left(z,r\right)\;and\;cap\left(A\right)>CT^{\frac{\left(d-2)(1-\epsilon \right)}{2}}\right)\\ \geq & 1-\exp\{-C*u*r^{d-2}\}-\sum_{k=1}^{n_{0}}\exp\left(-C*\min\left\{u^{k}*{\left(F\left(d,T^{1-\epsilon }\right)\right)}^{k+1},T^{\frac{\left(d-2)(1-\epsilon \right)}{2}}\right\}\right)\\ & -\left(n_{0}+1\right)\exp\left(-CT^{0.5\epsilon }\right). \end{array}$$

Meanwhile, by Lemma 8 of [11], we can ensure that the connected cluster mentioned above is contained by $B(z,r+T^{0.5+0.5\epsilon })$ with a probability of at least $1-C^{\prime }\exp\left(-CT^{\gamma }\right)$ for some γ>0. In conclusion, for d=3,

(29) $$P\left(E\right)\geq 1-exp\left(-C*ur\right)-\exp\left(-CT^{0.5\epsilon }\right)-C^{\prime }\exp\left(-CT^{\gamma }\right),$$

and for d≥4,

(30) $$\begin{array}{cc} P(E)\geq & 1-\exp\{-C*u*r^{d-2}\}-\sum_{k=1}^{n_{0}}\exp\left(-C*\min\left\{u^{k}*{\left(F\left(d,T^{1-\epsilon }\right)\right)}^{k+1},T^{\frac{\left(d-2)(1-\epsilon \right)}{2}}\right\}\right)\\ & -\left(n_{0}+1\right)\exp\left(-CT^{0.5\epsilon }\right)-C^{\prime }\exp\left(-CT^{\gamma }\right). \end{array}$$

For *F*, by Lemma 3.4 of [12], we have that there exists constants $C,\gamma^{\prime }>0$ such that

(31) $$P_{0}\left(\max\{\right|X_{i}|:0\leq i\leq T\}\geq T^{0.5+0.5\epsilon })\leq C\exp\{-T^{\gamma^{\prime }}\}.$$

Then, by (31), Lemma 3.1 of [6], and the approach in Lemma 12 of [11], we have

(32) $$P\left(F\right)\geq 1-C^{\prime }R^{2d}*\exp\left(-C*u*R^{2-d}*T^{\left(d-2)(1-\epsilon \right)}\right).$$

For event *G*, for any $x\in Z^{d}\backslash B\left(2R\right)$, since $P_{x}^{\left(T\right)}\left(traversing\;B\left(1.5R\right)\right)\leq {\left(1-\frac{1}{T+1}\right)}^{|x|-1.5R}$, we have

(33) $$\begin{array}{cc} \; & P^{u,T}\left(\exists path\;starting\;from\;Z^{d}\backslash B\left(2R\right)\;and\;intersecting\;B\left(1.5R\right)\right)\\ \leq & \sum_{|x|>2R}1-\exp\left(-\frac{2du}{T+1}*{\left(1-\frac{1}{T+1}\right)}^{|x|-1.5R}\right)\\ \leq & \sum_{|x|>2R}\frac{2d*m_{0}}{T+1}*{\left(1-\frac{1}{T+1}\right)}^{|x|-1.5R}\leq C^{\prime }*\exp\left(-CT^{\epsilon }\right). \end{array}$$

For the remaining subevents of *G*, the estimates are similar. In conclusion,

(34) $$P\left(G^{c}\right)\leq C^{\prime \prime }*\exp\left(-CT^{\epsilon }\right).$$

Finally, by (29), (30), (32), and (34), it is elementary to check that when $M(d,\delta ,m_{0})$ is large enough, events *E*, *F*, and *G* all happen with sufficiently high probability.

At the same time, we also have the following result on the subcritical phase when *u* is large. This, together with Claim (iv) of Theorem 3, characterize the asymptotic order of the phase diagram.

**Proposition** **2.** *For d≥3, there exists $c_{0}\left(d\right)>0$ and $u_{0}\left(d\right)>0$ such that for any $u>u_{0}\left(d\right)$ and $0<T<\frac{c_{0}\left(d\right)}{u}$, ${\mathrm{FI}}_{d}^{u,T}$ does not have an infinite cluster almost surely.*

**Proof.**  This proposition is a direct corollary of the proof of Theorem 2 [5]. By Section 7 of [5], it has been proved that for any $0<T\leq T_{0}$, ${\mathrm{FI}}_{d}^{u,T}$ does not have an infinite cluster a.s. if $T_{0}$ satisfies the following two conditions:

1. $6dT_{0}<1$;
2. ${\left(\frac{1-T_{0}}{1-6dT_{0}}\right)}^{\left\lceil 2de*u+\log(3d)\right\rceil }\leq 2.$

For condition 2, it is sufficient to have

$$\log\left(\frac{1-T_{0}}{1-6dT_{0}}\right)\leq \frac{\log(2)}{2de*u+\log(3d)+1}.$$

Note that $\log\left(\frac{1-T_{0}}{1-6dT_{0}}\right)=\log\left(1+\frac{\left(6d-1\right)T_{0}}{1-6dT_{0}}\right)\leq \frac{\left(6d-1\right)T_{0}}{1-6dT_{0}}$. In order to have $\frac{\left(6d-1\right)T_{0}}{1-6dT_{0}}\leq \frac{\log(2)}{2de*u+\log(3d)+1}$, we only need

(35) $$2de\left(6d-1\right)*uT_{0}+\left[6d\log(2)+(\log(3d)+1)(6d-1)\right]T_{0}\leq \log\left(2\right).$$

In conclusion, if we take $c_{0}\left(d\right)=\frac{\log(2)}{4de(6d-1)}$ and $u_{0}\left(d\right)=c_{0}*\max\left\{6d+1,\frac{2\left[6d\log(2)+(\log(3d)+1)(6d-1)\right]}{\log(2)}\right\}$, then condition 1, 2 hold for any $u>u_{0}$ and $T_{0}=\frac{c_{0}\left(d\right)}{u}$.  □

## 5. Numerical Exploration on Phase Transition

The previous section provides (partial) characterizations on the super and subcritical phases of FRI, while it remains unknown whether there is a unique critical value $T_{c}$ such that FRI almost surely percolates when $T>T_{c}$ and has no infinite cluster when $T<T_{c}$. In this section, we make numerical explorations towards this direction. The general guidelines behind criteria of numerical tests in this section are mostly inspired by [13,14].

In order to investigate the existence and uniqueness of $T_{c}$, we develop the following parallel computing algorithm in order to efficiently sample the configuration within a large box in $Z^{d}$ of size *N*, with data transferred within up to 80 cores via the Message Passing Interface (MPI). In Section 4.1 of [5], it has been shown that we can sample the configuration of FRI restricted within an infinite set *K* by Algorithm 1:
**Algorithm 1** Finitary Random Interlacement.Divide the vertices x∈V into $N_{p}$ mutually independent batches $\left(B_{1}\left(N\right),\cdots ,B_{N_{p}}\left(N\right)\right)$ and distribute one batch to one processor.For *s*-th batch, for any $x\in B_{s}\left(N\right)$, sample an independent random variable $N_{x}∼Pois\left(\frac{2du}{T+1}\right)$. Then, sample a sequence of i.i.d. random walks ${\left\{\eta_{i}\right\}}_{i=1}^{N_{x}}$ independent to $N_{x}$, with distribution $P_{x}^{\left(T\right)}$.For each trajectory $\eta_{i},\;i\leq N_{x}$ of the random walk mentioned above, if η escapes from *K* (i.e., for any n≥1, η(n)∉K), then start a new independent random walk with distribution $P_{x}^{\left(T\right)}$ and collect its trajectory ${\hat{\eta }}_{i}$; if not, jump to the next trajectory $\eta_{i+1}$.Collect all the trajectories $\underset{s,i}{⋃}{\hat{\eta }}_{i}\cap K$ from all processors.

**Figure 4:**
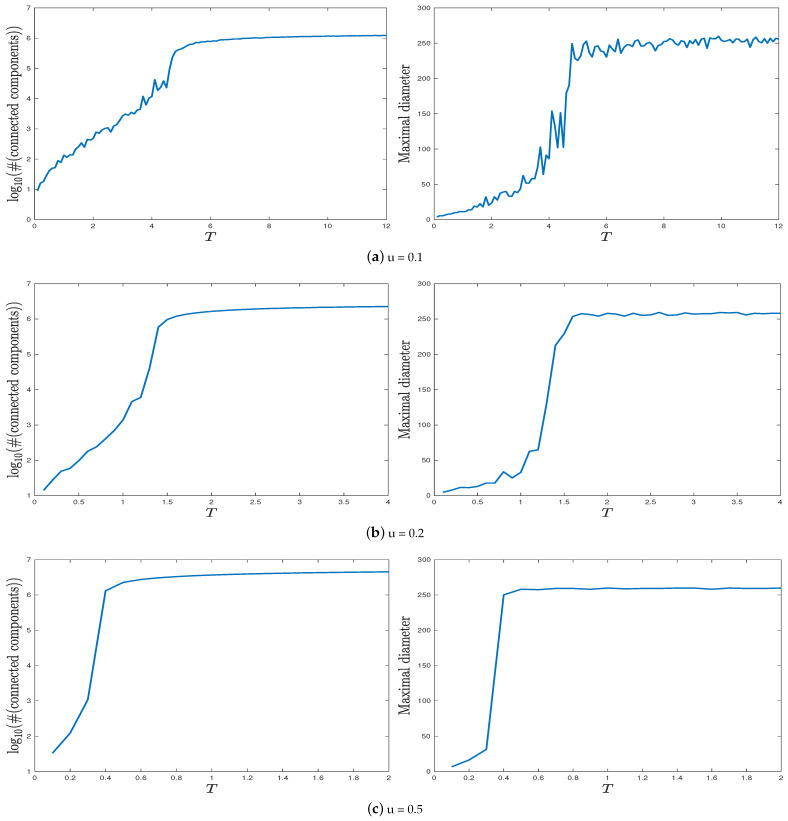
Stochastic simulations on the sizes (left, in logarithm scale) and maximal diameters (right) of FRI largest connected components. Phase transitions are clearly observed for different *u*. (**a**) u = 0.1; (**b**) u = 0.2; (**c**) u = 0.5.

Since $\underset{s,i}{⋃}{\hat{\eta }}_{i}\cap K$ is identically distributed as the collections of all fibers in ${\mathrm{FI}}_{d}^{u,T}$ which traverse *K*, we have that $\underset{s,i}{⋃}{\hat{\eta }}_{i}\cap K\overset{d}{=}{\mathrm{FI}}_{d}^{u,T}\cap K$. Using the aforementioned algorithm, one can naturally look at the size (in either cardinality or diameter) of the largest connected component within a large box in $Z^{d}$ of size *N*, say ${\left[0,N\right]}^{d}\cap Z^{d}$. In the supercritical phase, there should be a macroscopic largest connected component within ${\left[0,N\right]}^{d}\cap Z^{d}$, since it should, with high probability, be the largest cluster in the intersection(s) between the infinite cluster and our box. Meanwhile, in the subcritical phase, the largest connected component should be microscopic with respect to *N*. See Figure 1a for illustration. In Figure 4, we present stochastic simulation results on the cardinality and maximal diameter of the FRI’s largest connected components within ${\left[0,N\right]}^{3}\cap Z^{3}$, for N=150, u=0.1,0.2,0.5, and various *T*’s, under only one realization. In order to manifest the phase transition more clearly, we choose different ranges of *T* under different *u*. Although the curves are not smooth due to some random fluctuations and size effects, numerical evidences seem to strongly support the existence and uniqueness of $T_{c}$, which seems to be smaller as *u* becomes larger.

**Figure 5:**
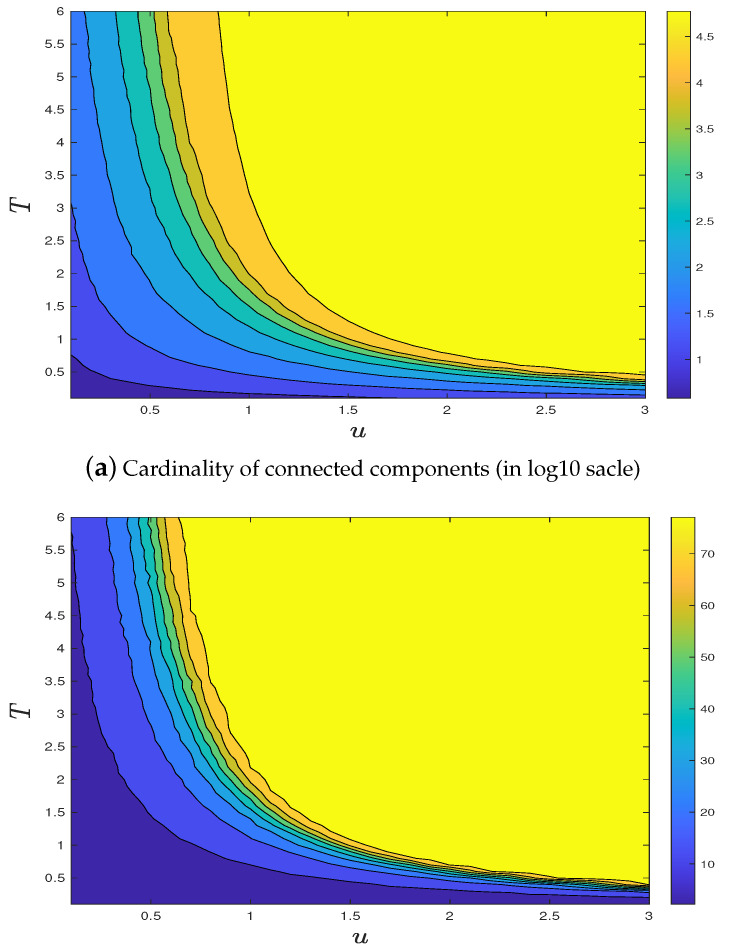
Illustration for the existence of phase diagram of FRI largest connected components. Each point is obtained by the averaging of 100 i.i.d FRIs. (**a**) Cardinality of connected components (in log10 scale); (**b**) maximal diameter.

Figure 4 strongly supports the existence of a unique critical fiber length, at least for the *u*’s we chose. This encourages us to extend the test for all combinations of (u,T)’s within an appropriate grid [0,3]×[0,6] with Δu=0.1, ΔT=0.1. Noting that the computational cost grows as $O(N^{6})$, we need to work on a smaller N=50. In order to avoid the extra randomness due to the smaller box size, we run 100 i.i.d. FRI copies for each combination of (u,T) and approximate the expected size of the largest cluster. Results shown in Figure 5 indicate that the existence and uniqueness of critical fiber length seem to hold for all *u*’s.

Based on the aforementioned numerical evidences, we propose the following conjecture:

**Conjecture** **1.** *For all* d≥3 *and* u>0 *, there is a* $T_{c}=T_{c}\left(u\right)\in \left(0,\infty \right)$ *such that for* ${\mathrm{FI}}_{d}^{u,T}$

- *There is a.s. no infinite cluster for all*$T<T_{c}$.
- *There is a.s. a unique infinite cluster for all*$T>T_{c}$.

**Remark** **8.** *Part of Conjecture 1 is also briefly mentioned in a revised version of [5] without further study on (non)monotonicity or numerical evidences.*

**Figure 6:**
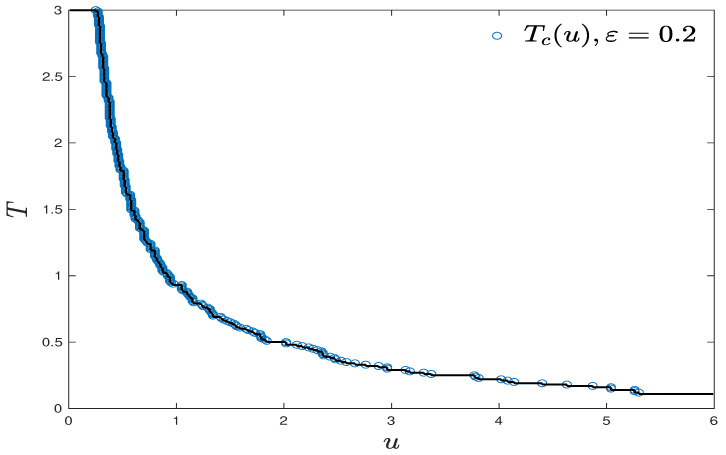
A more precise estimation of the curve of $T_{c}$ via the hill climbing algorithm, N=50,ε=0.2.

If we for now accept the existence of critical value in the conjecture above, we then explore the shape and asymptotic of the curve of $T_{c}$. With the help of monotonicity over *u* (Claim (i) Theorem 3), we use the following hill climbing algorithm (Algorithm 2) and record the ascending path $\left(u_{0},T_{0}\right),\cdots ,\left(u_{n},T_{n}\right),\cdots$, with small spacing ΔT=0.01, Δu=0.01, $u_{0}=3$, $T_{0}=0.01$. This algorithm significantly reduces the numerical costs in finding the boundary of phase transitions (as shown in Figure 6).

**Figure 7:**
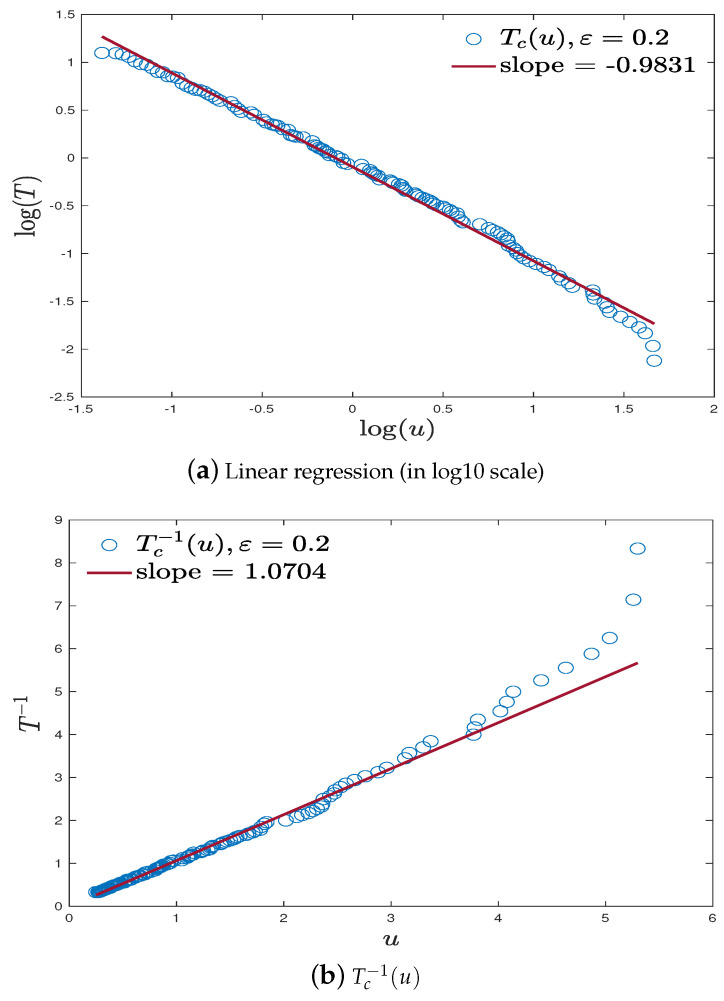
Linear regression on $\left(u,T_{c}\left(u\right)\right)$ (in logarithm scale) and $\left(u,T_{c}^{-1}\left(u\right)\right)$, ϵ=0.2. (**a**) Linear regression (in log10 scale); (**b**) $T_{c}^{-1}\left(u\right)$.

A linear regression on $\left(\log u,\log\left(T_{c}\left(u\right)\right)\right)$ (marked by circle), with N=50,ε=0.2 is shown in Figure 7a. In addition, linear regression on $\left(u,T_{c}^{-1}\left(u\right)\right)$ in Figure 7b seems to indicate that the exponent in the upper bound of $T_{c}$ in Claim (v) Theorem 3 is close to 1. This observation together with the theoretical findings in Theorem 3 and Proposition 2 motivates us to propose the following conjecture:

**Conjecture** **2.** *For all*d≥3,
**Algorithm 2** Hill climbing algorithm.**Input:** The box size *N*, A sufficiently small initial $T_{0}$, a sufficiently large initial $u_{0}$, spacings ΔT and Δu and a threshold ε.Start from n=0 and $\left(u_{0},T_{0}\right)$.Run one realization of FRI under the parameters $\left(u_{n},T_{n}\right)$ and calculate the maximal diameter $d_{n}$ of the largest cluster of such FRI.If $d_{n}<\sqrt{3}\varepsilon N$, $\left(u_{n+1},T_{n+1}\right)=\left(u_{n},T_{n}+\Delta T\right)$, go to Step 5.If $d_{n}\geq \sqrt{3}\varepsilon N$, $\left(u_{n+1},T_{n+1}\right)=\left(u_{n}-\Delta u,T_{n}\right)$ and mark it by circle, go to Step 5.Terminate when $u_{n+1}<0$, otherwise go back to Step 2 with parameters $\left(u_{n+1},T_{n+1}\right)$. **Output:** The path $\left(u_{0},T_{0}\right),\cdots ,\left(u_{n},T_{n}\right),\cdots$

- *there is a constant*$c_{d}$*such that*$\lim_{u\to 0^{+}}uT_{c}\left(u\right)=c_{d}$;
- *Since it is very unlikely for a fiber to run more than one step when T is small, we conjecture that the upper bound of*$T_{c}$*in Claim (iv) Theorem 3 is sharp, i.e.,*
  $$\lim_{u\to \infty }uT_{c}\left(u\right)=\frac{-\log(1-p_{d}^{c})}{2}.$$

**Remark** **9.** *It is worth noting that, as a result of the finite size effect of box size N, the estimated slope in linear regression under logarithm scale can be sensitive with respect to ϵ. We found in simulation that slope equals to −1.48 when ϵ=0.1, −0.98 when ϵ=0.2, −0.82 when ϵ=0.3, −0.76 when ϵ=0.4, and −0.73 when ϵ=0.5. Recalling the theoretical upper bound of $T_{c}$ in Claim (iv) Theorem 3, and lower bound in Proposition 2, it is not hard to prove that slopes converge to −1, as N→∞ for all $\epsilon \in (0,1/\sqrt{3})$. So, for N=50, it seems the phase diagram has best precision when ϵ≈0.2. Thus, we plot $\left(u,T_{c}^{-1}\left(u\right)\right)$ under this setting and find that the slope in linear regression is 1.07, which is very close to theoretical value 1. Here, we only use 112 points (u≤4) for linear regression and the remaining 9 points (u>4) seem to deviate from the line. The possible reason is that $T_{c}$ is very small for large u and thus might not be precisely captured.*

## Appendix A. Proof of Lemma 3

In the following arguments, we need a more precise construction of SRW on $Z^{d}$: For 1≤i≤d, let ${\left\{X_{n}^{i}\right\}}_{n=1}^{\infty }$ be an i.i.d. sequence of random variables with distribution $P\left(X_{n}^{i}=-1\right)=P\left(X_{n}^{i}=1\right)=\frac{1}{2}$. Then, ${\left\{S_{n}=\sum_{k=1}^{n}X_{k}^{i}\right\}}_{n=0}^{\infty }$ forms *d* independent copies of 1-dimension SRW’s. We also define an i.i.d. sequence of random variables ${\left\{D_{n}\right\}}_{n=1}^{\infty }$ with distribution $P\left(D_{n}=j\right)=\frac{1}{d}$, 1≤j≤d. Then, $S_{n}^{d}=\sum_{k=1}^{n}X_{k}^{D_{k}}e_{D_{k}}$ is an SRW on $Z^{d}$.

Before we prove Lemma 3, there are some preparations.

**Lemma** **A1.**
*For any $n_{0}>d^{1.5}$, consider a stopping time $\bar{\Gamma }:=\inf\left\{n\geq 0:\exists 1\leq i\leq d,|S_{\tau_{n}^{\left(i\right)}}^{d,i}|\geq n_{0}^{\frac{1}{8}}\right\}$, where $S_{m}^{d,i}=\sum_{k=1}^{m}X_{k}^{i}e_{i}$ and $\tau_{n}^{\left(i\right)}=\left|\left\{1\leq k\leq n:D_{k}=i\right\}\right|$ for 1≤i≤d. Then, there exists δ>0 such that for any sufficiently large $n_{0}$,*

(A1) $$P_{0}\left(\bar{\Gamma }>n_{0}\right)\leq d*\exp\left(-\delta n_{0}^{\frac{1}{12}}\right).$$

**Proof.** By the invariance principle, we have for any l≥1,

(A2) $$\lim_{n_{0}\to \infty }P_{0}\left(\left|S_{ln_{0}^{\frac{1}{4}}}^{d,1}-S_{\left(l-1\right)n_{0}^{\frac{1}{4}}}^{d,1}\right|\leq 2n_{0}^{\frac{1}{8}}\right)=P\left(B_{1}\leq 2\right)<1,$$

where ${\left\{B_{t}\right\}}_{t\geq 0}$ is a Brownian Motion starting from 0. Therefore, there exists δ>0 such that for all sufficient large $n_{0}$,

(A3) $$P_{0}\left(\left|S_{ln_{0}^{\frac{1}{4}}}^{d,1}-S_{\left(l-1\right)n_{0}^{\frac{1}{4}}}^{d,1}\right|\leq 2n_{0}^{\frac{1}{8}}\right)<e^{-\delta }.$$

Obviously, since $n_{0}>d^{1.5}$, there must exist $j_{0}\in \left\{1,2,\cdots ,d\right\}$ such that $|\left\{1\leq i\leq n_{0}:D_{i}=j_{0}\right\}|>\frac{n_{0}}{d}>n_{0}^{\frac{1}{3}}$. By (A3) and symmetry, we have

(A4) $$\begin{array}{cc} P_{0}\left(\bar{\Gamma }>n_{0}\right)\leq & \sum_{k=1}^{d}P_{0}\left(|\left\{1\leq i\leq n_{0}:D_{i}=j_{0}\right\}|>n_{0}^{\frac{1}{3}},\max_{j\leq n_{0}^{\frac{1}{3}}}\left|\left\{S_{j}^{d,k}\right\}\right|\leq n_{0}^{\frac{1}{8}}\right)\\ \leq & d*P_{0}\left(\overset{n_{0}^{\frac{1}{12}}}{\underset{l=1}{⋂}}\left\{\left|S_{ln_{0}^{\frac{1}{4}}}^{d,1}-S_{\left(l-1\right)n_{0}^{\frac{1}{4}}}^{d,1}\right|\leq 2n_{0}^{\frac{1}{8}}\right\}\right)\\ = & d*\prod_{l=1}^{n_{0}^{\frac{1}{12}}}P_{0}\left(\left|S_{ln_{0}^{\frac{1}{4}}}^{d,1}-S_{\left(l-1\right)n_{0}^{\frac{1}{4}}}^{d,1}\right|\leq 2n_{0}^{\frac{1}{8}}\right)\leq d*\exp\left(-\delta n_{0}^{\frac{1}{12}}\right). \end{array}$$

□

**Lemma** **A2.**
*There exists c>0 (c is independent to d) such that for any d≥20 and any $x_{0}\in Z^{d}$ such that ∃1≤i≤d, $x_{0}^{\left(i\right)}>n_{0}^{\frac{1}{8}}$,*

(A5) $$P_{x_{0}}\left(H_{d,\{0\}}<\infty \right)\leq cn_{0}^{-2}.$$

**Proof.** Without loss of generality, we assume that $i\leq \frac{d}{2}$. Then, we define $I_{l}=\max\left\{1,i-9\right\}$, $I_{r}=\max\left\{19,i+9\right\}$ and ${\hat{x}}_{0}=\left(x_{0}^{\left(I_{l}\right)},x_{0}^{\left(I_{l}+1\right)},..,x_{0}^{\left(I_{r}\right)}\right)\in Z^{19}$, where $\left|\right|{\hat{x}}_{0}{\left|\right|}_{2}\geq n_{0}^{\frac{1}{8}}$. We define a stopping time ${\hat{H}}_{d,\{0\}}=\inf\left\{n\geq 0:S_{\tau_{n}^{\left(i\right)}}^{d,i}=0,\forall i\in \left[I_{l},I_{r}\right]\right\}$. It is easy to see that ${\hat{H}}_{d,\{0\}}\leq H_{d,\{0\}}$. By Proposition 6.5.1 of [9], we have

(A6) $$P_{x_{0}}\left(H_{d,\{0\}}<\infty \right)\leq P_{x_{0}}\left({\hat{H}}_{d,\{0\}}<\infty \right)=P_{{\hat{x}}_{0}}\left(H_{19,\{0\}}<\infty \right)\leq c{\left(n_{0}^{\frac{1}{8}}\right)}^{-(19-2)}\leq cn_{0}^{-2}.$$

□

Now, we are able to prove Lemma 3.

**Proof** **of** **Lemma** **3.** Since $\left\{n\leq H_{d,\{0,x_{1}\}}<\infty \right\}\subset \left\{n\leq H_{d,\{0\}}<\infty \right\}\cup \left\{n\leq H_{d,\{x_{1}\}}<\infty \right\}$ for any integer n≥1, we have

(A7) $$\begin{array}{c} R_{d}\leq \sum_{n=1}^{\infty }\left[P_{0}\left(n\leq H_{d,\{0\}}<\infty \right)+P_{0}\left(n\leq H_{d,\{x_{1}\}}<\infty \right)\right]. \end{array}$$

By symmetry, we have for any n≥1,

(A8) $$P_{0}\left(n+1\leq H_{d,\{0\}}<\infty \right)=\frac{1}{2d}\sum_{x\in Z^{d}{,||x||}_{1}=1}P_{x}\left(n\leq H_{d,\{0\}}<\infty \right)=P_{0}\left(n\leq H_{d,\{x_{1}\}}<\infty \right).$$

Combine (A7) and (A8),

(A9) $$\begin{array}{cc} R_{d}\leq & 2\sum_{n=1}^{\infty }P_{0}\left(n\leq H_{d,\{0\}}<\infty \right)\\ = & 2\left[2P_{0}\left(2\leq H_{d,\{0\}}<\infty \right)+\sum_{n=3}^{d^{1.5}}P_{0}\left(n\leq H_{d,\{0\}}<\infty \right)+\sum_{n=d^{1.5}+1}^{\infty }P_{0}\left(n\leq H_{d,\{0\}}<\infty \right)\right]. \end{array}$$

By the corollary of (1.14) in [15], there exists c>0 such that for all sufficient large *d*,

(A10) $$P_{0}\left(H_{d,\{0\}}<\infty \right)\leq \frac{1}{2d}+\frac{c}{d^{2}}.$$

Note that $P_{0}\left(H_{d,\{0\}}=1\right)=0$ and $P_{0}\left(H_{d,\{0\}}=2\right)=\frac{1}{2d}$. By (A10), we have

(A11) $$P_{0}\left(3\leq H_{d,\{0\}}<\infty \right)\leq \frac{c}{d^{2}}.$$

Therefore, for the first and second term on the RHS of (A9), we have

(A12) $$2P_{0}\left(2\leq H_{d,\{0\}}<\infty \right)+\sum_{n=3}^{d^{1.5}}P_{0}\left(n\leq H_{d,\{0\}}<\infty \right)\leq \frac{1}{d}+\frac{c}{d^{0.5}}.$$

For the last term on the RHS of (A9), by Lemma A1, Lemma A2, and the strong Markov property, we have

(A13) $$\begin{array}{cc} \sum_{n=d^{1.5}+1}^{\infty }P_{0}\left(n\leq H_{d,\{0\}}<\infty \right)\leq & \sum_{n=d^{1.5}+1}^{\infty }\left[P_{0}\left(\bar{\Gamma }\leq n,n\leq H_{d,\{0\}}<\infty \right)+P_{0}\left(\bar{\Gamma }>n\right)\right]\\ \leq & \sum_{n=d^{1.5}+1}^{\infty }\left[cn^{-2}+d*e^{-\delta n^{\frac{1}{12}}}\right]\leq c^{\prime }\left(d^{-1.5}+d*e^{-\delta d^{\frac{1}{8}}}\right). \end{array}$$

Combine (A9), (A12), and (A13),

(A14) $$R_{d}\leq 2\left[\frac{1}{d}+\frac{c}{d^{0.5}}+c^{\prime }\left(d^{-1.5}+d*e^{-\delta d^{\frac{1}{8}}}\right)\right].$$

Since the RHS of (A14) converges to 0 as d→∞, we finally get $\lim_{d\to \infty }R_{d}=0$. □

## Appendix B. Proof of Theorem 2

By Theorem 12.2 of [16] and the remark about ”finite energy“ in it, in order to prove that ∀d≥3, ∀u>0, ∀T>0, ${\mathrm{FI}}_{d}^{u,T}$ (note that ${\mathrm{FI}}_{d}^{u,T}$ is considered as a bond percolation on $Z^{d}$ here) has one infinite cluster at most almost surely, it is sufficient to confirm two conditions:

1. ${\mathrm{FI}}_{d}^{u,T}$ is translation invariant;
2. ${\mathrm{FI}}_{d}^{u,T}$ has insertion tolerance and deletion tolerance.

For FRI, translation invariance is an elementary property, so we just focus on insertion tolerance and deletion tolerance. The precise definitions are stated here.

**Definition** **A1** (Definition 3.2, [17]).

1. *(insertion tolerance) Let $L^{d}$ be the set of all edges in $Z^{d}$. For any event $A\subset {\left\{0,1\right\}}^{L^{d}}$ and any edge $e\in L^{d}$, define a mapping $\Pi_{e}^{+}$: $A\to \Pi_{e}^{+}\left(A\right):=\left\{A\cup \{e\}:A\in A\right\}$. Say a bond percolation (its law is denoted by P) has insertion tolerance if ∀ event $A\subset {\left\{0,1\right\}}^{L^{d}}$, $\forall e\in L^{d}$,*
  $$P\left(A\right)>0⟹P\left(\Pi_{e}^{+}\left(A\right)\right)>0.$$
2. *(deletion tolerance) Let $\Pi_{e}^{-}$: $A\to \Pi_{e}^{-}\left(A\right):=\left\{A\backslash \{e\}:A\in A\right\}$. Then, say a bond percolation with law P has deletion tolerance if* ∀ *event $A\subset {\left\{0,1\right\}}^{L^{d}}$, $\forall e\in L^{d}$,*
  $$P\left(A\right)>0⟹P\left(\Pi_{e}^{-}\left(A\right)\right)>0.$$

### Appendix B.1. Insertion Tolerance

By translation invariance, it is sufficient to prove for $e=e_{1}=\left\{0,x_{1}\right\}$, where $x_{1}=\left(1,0,\cdots ,0\right)\in Z^{d}$.

We have the canonical mapping π: $\omega =\sum_{i=1}^{\infty }\delta_{\eta_{i}}\to A\in {\left\{0,1\right\}}^{L^{d}}$, where $A\left(e\right)=1-\prod_{i=1}^{\infty }{𝟙}_{e\notin \eta_{i}}$ for any $e\in L^{d}$. Then, we have $P=P^{u,T}\circ \pi^{-1}$. Without causing confusion, for any collection *I* of paths in $W^{\left[0,\infty \right)}$, *I* can be regarded as a point measure, so we also define π(I) in the same way.

For any event $A\subset {\left\{0,1\right\}}^{L^{d}}$ such that $P\left(A\right)>0$, then we have $P\left(A\cap \{e_{1}\;is\;open\}\right)>0$ or $P\left(A\cap \{e_{1}\;is\;close\}\right)>0$. If $P\left(A\cap \{e_{1}\;is\;open\}\right)>0$, then $P\left(\Pi_{e_{1}}^{+}\left(A\cap \{e_{1}\;is\;open\}\right)\right)=P\left(A\cap \{e_{1}\;is\;open\}\right)>0$. Note that $\Pi_{e_{1}}^{+}\left(A\cap \{e_{1}\;is\;open\}\right)\subset \Pi_{e_{1}}^{+}\left(A\right)$, we have $P\left(\Pi_{e_{1}}^{+}\left(A\right)\right)>0$.

Meanwhile, if $P\left(A\cap \{e_{1}\;is\;open\}\right)=0$ and $P\left(A\cap \{e_{1}\;is\;close\}\right)>0$, let $B=A\cap \{e_{1}\;is\;close\}$. Denote by $S_{e{}_{1}}$ the collection of all paths with first step $e_{1}$ and by $S_{e{}_{1}}^{c}$ the collection of paths starting from $Z^{d}\backslash \left\{0,x_{1}\right\}$ and the paths starting from $\left\{0,x_{1}\right\}$ whose first steps are not $e_{1}$. By definition of FRI and property of Poisson distribution, we know that $S_{e{}_{1}}$ and $S_{e{}_{1}}^{c}$ are independent. Note that for any $\omega =\sum_{i=1}^{n}\delta_{\eta_{i}}\in \pi^{-1}\left(B\right)$, we have ∀i≥1, $\eta_{i}\in S_{e{}_{1}}^{c}$. Therefore,

(A15) $$P\left(B\right)=P^{u,T}\left(\pi^{-1}\left(B\right)\right)=P^{u,T}\left(S_{e{}_{1}}=\emptyset \right)*P^{u,T}\left[\omega :\pi \left(\sum_{i=1}^{\infty }\delta_{\eta_{i}}\cdot {𝟙}_{\eta_{i}\in S_{e{}_{1}}^{c}}\right)\in B\right].$$

Let $\eta *=\left(0,x_{1}\right)\in W_{d}^{\left[0,\infty \right)}$ and then we have $\left\{\omega +\delta_{\eta^{*}}:\omega \in \pi^{-1}\left(B\right)\right\}\subset \Pi_{e_{1}}^{+}\left(B\right)$. Thus,

(A16) $$\begin{array}{cc} P^{u,T}\left(\left\{\omega +\delta_{\eta^{*}}:\omega \in \pi^{-1}\left(B\right)\right\}\right)= & P^{u,T}\left(S_{e{}_{1}}=\left\{\eta^{*}\right\}\right)*P^{u,T}\left[\omega :\pi \left(\sum_{i=1}^{\infty }\delta_{\eta_{i}}\cdot {𝟙}_{\eta_{i}\in S_{e{}_{1}}^{c}}\right)\in B\right]\\ = & P^{u,T}\left(\pi^{-1}\left(B\right)\right)*\frac{P^{u,T}\left(S_{e{}_{1}}=\left\{\eta^{*}\right\}\right)}{P^{u,T}\left(S_{e{}_{1}}=\emptyset \right)}>0. \end{array}$$

Therefore,

(A17) $$P\left(\Pi_{e_{1}}^{+}\left(A\right)\right)\geq P\left(\Pi_{e_{1}}^{+}\left(B\right)\right)\geq P^{u,T}\left(\left\{\omega +\delta_{\eta^{*}}:\omega \in \pi^{-1}\left(B\right)\right\}\right)>0.$$

In conclusion, $P\left(A\right)>0⟹P\left(\Pi_{e}^{+}\left(A\right)\right)>0$, which means ${\mathrm{FI}}_{d}^{u,T}$ has insertion tolerance.

### Appendix B.2. Deletion Tolerance

Similarly, if $P\left(A\cap \{e_{1}\;is\;close\}\right)>0$, then

$$P\left(\Pi_{e_{1}}^{-}\left(A\right)\right)\geq P\left(\Pi_{e_{1}}^{-}\left(A\cap \{e_{1}\;is\;close\}\right)\right)=P\left(A\cap \{e_{1}\;is\;close\}\right)>0.$$

On the other hand, if $P\left(A\cap \{e_{1}\;is\;open\}\right)>0$, let $C=A\cap \{e_{1}\;is\;open\}$. Denote the collection of all paths traversing $e_{1}$ by ${\hat{S}}_{e_{1}}$ and the number of paths in ${\hat{S}}_{e_{1}}$ by $N_{e_{1}}$. Then, there exists $N\in N^{+}$ such that

(A18) $$P^{u,T}\left(\pi^{-1}\left(C\right),N_{e_{1}}=N\right)>0.$$

Since $W^{\left[0,\infty \right)}$ is countable, there exists $\zeta_{1},\cdots ,\zeta_{N}\in W_{d}^{\left[0,\infty \right)}$ such that

(A19) $$P^{u,T}\left(\pi^{-1}\left(C\right),{\hat{S}}_{e_{1}}=\left\{\zeta_{1},\cdots ,\zeta_{N}\right\}\right)>0.$$

Let $D=\left\{\zeta_{1}\left(0\right),\cdots ,\zeta_{N}\left(0\right)\right\}\cup \left\{0,x_{1}\right\}\subset Z^{d}$. By (A19), there exists a fixed trajectory Ξ of the set of all paths starting from *D* (note that $\zeta_{1},\cdots ,\zeta_{N}\in \Xi$) such that

(A20) $$P^{u,T}\left(\left\{\omega =\sum_{i=1}^{\infty }\delta_{\eta_{i}}:{\hat{S}}_{e_{1}}\subset S_{D}\left(w\right)=\Xi ,\Pi_{\pi (\Xi )}^{+}\circ \pi \left(\sum_{i=1}^{\infty }\delta_{\eta_{i}}\cdot {𝟙}_{\eta_{i}\left(0\right)\notin D}\right)\in C\right\}\right)>0,$$

where $S_{D}\left(w\right)$ is the collection of all paths starting from *D* in ω and for any $E\in {\left\{0,1\right\}}^{L^{d}}$ such that only finite edges are open (denote them by $\left\{\rho_{1},\cdots ,\rho_{m}\right\}$), $\Pi_{E}^{+}:=\Pi_{\rho_{m}}^{+}\circ \cdots \circ \Pi_{\rho_{1}}^{+}$.

Since the paths starting from *D* and $Z^{d}\backslash D$ are independent and the event $\left\{{\hat{S}}_{e_{1}}\subset S_{D}\left(w\right)\right\}=\left\{all\;the\;paths\;starting\;from\;Z^{d}\backslash D\;don^{\prime }t\;traverse\;e_{1}\right\}$, we have

(A21) $$P^{u,T}\left(S_{D}=\Xi \right)*P^{u,T}\left(\left\{\omega =\sum_{i=1}^{\infty }\delta_{\eta_{i}}:{\hat{S}}_{e_{1}}\subset S_{D}\left(w\right),\Pi_{\pi (\Xi )}^{+}\circ \pi \left(\sum_{i=1}^{\infty }\delta_{\eta_{i}}\cdot {𝟙}_{\eta_{i}\left(0\right)\notin D}\right)\in C\right\}\right)>0.$$

We define a mapping ϕ: for any $\eta \in W_{d}^{\left[0,\infty \right)}$, if $e_{1}\notin \eta$, set ϕ(η)={η}; if $e_{1}\in \eta =\left(\eta \left(0\right),\cdots ,\eta \left(n\right)\right)$, assume that $\left\{\eta \left(m\right),\eta \left(m+1\right)\right\}=e_{1}$ if and only if $m\in \{n_{1},..,n_{l}\}$, then $\phi \left(\eta \right)=\left\{\zeta_{0},\cdots ,\zeta_{l}\right\}\subset W_{d}^{\left[0,\infty \right)}$, where $\zeta_{0}=\left(\eta \left(0\right),\cdots ,\eta \left(n_{1}\right)\right)$ and for 1≤j≤l−1, $\zeta_{j}=\left(\eta \left(n_{j}+1\right),\cdots ,\eta \left(n_{j+1}\right)\right)$, $\zeta_{n_{l}}=\left(\eta \left(n_{l}+1\right),\cdots ,\eta \left(n\right)\right)$.

Denote that $\hat{\Xi }=\underset{\eta \in \Xi }{⋃}\phi \left(\eta \right)=\left\{\sigma_{1},\cdots ,\sigma_{M}\right\}$. Note that

$$\left\{\omega +\sum_{j=1}^{M}\delta_{\sigma_{j}}:\forall \eta_{i}\left(0\right)\notin D,\forall \eta_{i}\cap e_{1}=\emptyset ,\Pi_{\pi (\Xi )}^{+}\circ \pi \left(\sum_{i=1}^{\infty }\delta_{\eta_{i}}\cdot {𝟙}_{\eta_{i}\left(0\right)\notin D}\right)\in C\right\}\subset \pi^{-1}\left(\Pi_{e_{1}}^{-}\left(C\right)\right).$$

Therefore,

(A22) $$\begin{array}{cc} P\left(\Pi_{e_{1}}^{-}\left(A\right)\right)\geq & P\left(\Pi_{e_{1}}^{-}\left(C\right)\right)\\ \geq & P^{u,T}\left(S_{D}=\hat{\Xi }\right)P^{u,T}\\ & \left(\left\{\omega =\sum_{i=1}^{\infty }\delta_{\eta_{i}}:{\hat{S}}_{e_{1}}\subset S_{D}\left(w\right),\Pi_{\pi (\Xi )}^{+}\circ \pi \left(\sum_{i=1}^{\infty }\delta_{\eta_{i}}\cdot {𝟙}_{\eta_{i}\left(0\right)\notin D}\right)\in C\right\}\right)\\ > & 0. \end{array}$$

In conclusion, ${\mathrm{FI}}_{d}^{u,T}$ has deletion tolerance. By all the arguments above, the proof of Theorem 2 is completed.
